# Requirements for effective academic leadership in Iran: A Nominal Group Technique exercise

**DOI:** 10.1186/1472-6920-8-24

**Published:** 2008-04-22

**Authors:** Ali Bikmoradi, Mats Brommels, Alireza Shoghli, Zohreh Sohrabi, Italo Masiello

**Affiliations:** 1 Medical Management Centre, Department of Learning, Informatics, Management and Ethics, Karolinska Institutet, 171 77 Stockholm, Sweden; 2 Hamadan University of Medical sciences, Hamadan, Iran; 3 National Public Health Management Centre, Tabriz, Iran; 4 Department of Public health, University of Helsinki, Helsinki, Finland; 5 Department of Social Medicine, Zanjan Medical University of Sciences, Zanjan, Iran; 6 Iran University of Medical Sciences, Tehran, Iran; 7 Ministry of Health and Medical Education, Tehran, Iran

## Abstract

**Background:**

During the last two decades, medical education in Iran has shifted from elite to mass education, with a considerable increase in number of schools, faculties, and programs. Because of this transformation, it is a good case now to explore academic leadership in a non-western country. The objective of this study was to explore the views on effective academic leadership requirements held by key informants in Iran's medical education system.

**Methods:**

A nominal group study was conducted by strategic sampling in which participants were requested to discuss and report on requirements for academic leadership, suggestions and barriers. Written notes from the discussions were transcribed and subjected to content analysis.

**Results:**

Six themes of effective academic leadership emerged: 1)shared vision, goal, and strategy, 2) teaching and research leadership, 3) fair and efficient management, 4) mutual trust and respect, 5) development and recognition, and 6) transformational leadership. Current Iranian academic leadership suffers from lack of meritocracy, conservative leaders, politicization, bureaucracy, and belief in misconceptions.

**Conclusion:**

The structure of the Iranian medical university system is not supportive of effective academic leadership. However, participants' views on effective academic leadership are in line with what is also found in the western literature, that is, if the managers could create the premises for a supportive and transformational leadership, they could generate mutual trust and respect in academia and increase scientific production.

## Background

Effective leadership in medical university is a crucial component for overall success and an important requirement for a managerial position [1], and this is true for industry, non-profit and governmental organizations. However, the organizational complexity, multiple goals and traditional values of universities, promote ambiguous and contested leadership [2,3]. For example, many individuals holding managerial and administrative academic positions in medical universities, such as chancellor, vice-chancellor, dean, or head of department, are physicians or medical specialists without any preparation or training for academic leadership [1]. This may have negative consequences on the development of a successful university.

The Ministry of Health and Medical Education of Iran is responsible for public health, medical treatment and the management and planning of medical education, that is, training under supervision, of which expertise at all levels, from first degree to doctorate, is organized [4]. The medical education system in Iran has expanded very rapidly with a considerable number of schools, from 13 in 1979 to 47 in 2006 [5]. The number of faculties and programs has increased as well [6,7]. Significant steps have also been taken to improve the quality of health care delivery and medical education in this country [8]. For example, the Iranian government reorganized all managerial and provisional levels of medical education and health services. One major step was the gradual integration of medical education into health services with the purpose of health empowerment and a more relevant training for the workforce [6,9]. The integration sought also to provide a better response to the health needs of the community while broadening learning/teaching and research activities. However, the process of reform in the health service and medical education in Iran poses major challenges to faculty members and academic leaders.

Because of the complexity of Iranian medical universities, greater use should be made of qualitative data and analysis in the study of leadership, partly to gain a deeper understanding of the contextual issues associated with leadership [10]. This study explores, analyzes, and synthesizes the view held by experts on effective academic leadership requirements in Iranian medical education in order to prepare the groundwork for academic leadership development. One aim of this study is to assist potentially the Iranian Ministry of Health and Medical Education not only in preparing educational programs for the development of academic leadership but also in restructuring medical universities. This study was limited to medical universities with focus on the school of medicine.

## Methods

The Nominal Group Technique (NGT) [11] was used to identify key issues and consensus about appropriate management strategies. In June 2005 the researchers invited a number of experts to discuss the various requirements for effective academic leadership in medical universities. The meeting was held in Tehran, Iran.

### Participants

Participants were selected by consultation with the Ministry of Health and Medical Education. Written letters explaining the research goals and questions were sent to candidates. Originally, 40 persons acting in a variety of relevant positions and at different levels of expertise were invited. Only 24 were willing to meet on a selected date (three people with experience as deputies for education and research in the Ministry of Health, two chancellors, three vice-chancellors, two deans, three deputies for education in medical school, five department heads, three academic research centre heads, and three medical management specialists). All participants were experienced senior-level medical education managers, representing eight medical universities and three research centers in Iran.

### Working methods

Participants were grouped into three work teams representing university, school, and department levels respectively. To systematize the discussion, the participants were requested to discuss and report on requirements for three topics: 1) teaching and research leadership, 2) management and effective leadership, 3) faculties and effective leadership, their barriers, and suggestions for improvements. The results of each work team were presented as bullet points to all participants by the representative of each group during plenary sessions. This one-day meeting lasted for about 10 hours. Participants in each group initially wrote ideas relating to each topic at hand on paper and without talking to one another. Then, each individual, in a round-robin-like fashion, presented one idea from his or her list. A secretary wrote the idea on a flip chart in full view for the group. Discussions followed in order to clarify ideas or to express points of view. Group consensus was conducted by majority vote. The statements from all three work groups summed up to 100 bullet points as requirements for effective academic leadership and 68 barriers and suggestions for improvements. Consequently, the results were presented for everyone and a general consensus was reached about categorization, barriers and suggestions for improvements of effective academic leadership requirements in Iran.

### Analysis

Several methods exist for analyzing and interpreting qualitative data, as Kvale [12] describes, some of which include meaning condensation, meaning categorization, narrative structuring, meaning interpretation and generating meaning through *ad hoc *methods. The purpose of this study was to explore views for effective academic leadership requirements held by Iranian medical experts, and in accordance to this, we used a combination of meaning categorization and meaning interpretation methods.

### Ethical considerations

This study was conducted as part of a PhD dissertation at the Karolinska Institutet in Stockholm, Sweden, and as a joint project with the Ministry of Health and Medical Education of Iran which approved the study. All participants were informed of the purpose and design of the study and the voluntary nature of their participation.

## Results

The views of the expert panel members were grouped into six themes with 3–6 sub themes each (See Table 1). The themes are presented below.

**Table 1:** Effective academic leadership themes and subthemes

**Shared vision, goals, and strategies**	**Development and recognition performance**
-Strategic and autonomic approaches	-Efficient recognition system
-Considering globalization changes	-Job promotion
-Considering stakeholder's needs, demands, and expectations	-Promote capability, skill, and talent
**Teaching and research leadership**	**Fair and efficient management**
-Being prominent and role model	-Appropriate authority
-Continuous improvement	-Conflict management
-Establish efficient evaluation	-Law-oriented management
	-Efficient Reward system
	-Efficient monitoring system
	-Transparency and clarity
**Transformational leadership**	**Mutual trust, respect, and commitment**
-Collaboration and teamwork	-Interpersonal skills
-Networking	-Considering organizational culture
-Participation and delegation	-Achieving harmony
-Being diplomat	-Converging staff and organization needs
	-Leading ethics and values

### Shared vision, goals, and strategies

The participants' central idea was that academic work is facilitated through inspired and shared clear vision, goals, and strategies, consistently pursued and communicated with integrity, an understanding of individual needs, and energetic commitment. However, the participants usually expected to develop a vision by considering the community and stakeholder's needs, demands, and expectations and by also looking at globalization changes. In addition, visions, goals, and strategies were not perceived equally among faculties, staffs, and managers at departments, school, and university, emerging as unstable formalities.

### Teaching and research leadership

Another major view brought out was that of academic leaders who should be prominent, role models and oversee continuous improvement of research and teaching. It was discussed the crucial role of considering research, teaching, and health services as priorities, while adapting to macro-scale policies. Meanwhile, it was asserted that academic leadership needs to be established further. It also needed to develop a human resource network inside and outside the department, medical school and university and at the local, national, and international levels. These networks could create an effective academic leadership on learning, teaching, and research processes. The panelists also insisted on requirement nexus between applied research and community needs; basic and clinical sciences teaching and research; and department, school, and university with other organizations in society such as business and industry.

### Transformational and collaborative leadership

Panelists pointed out that academic leadership should be transformational and collaborative, emphasizing participation, delegation, and teamwork. Driven by the dynamic nature of the academic environment, recommendations for acceptable management based upon an increased utilization of teams and workgroups with multidisciplinary collaboration at local, regional, and international levels should offer a decreased reliance on traditional authority arrangements. It was proposed that academic leadership at all levels should enhance participation by delegating authorities and sharing responsibilities and decision making among heads of departments and faculty members. The medical education process needs a collaborative work spirit and leadership with full participation of leaders, faculty members and support staffs.

### Development and recognition performance

The experts highlighted that in order to succeed at new teaching, research, and leadership tasks, faculty development is essential. An effective and efficient reward system with appropriate and on time feedback should be able to improve output of academic work, according to possible results of an efficient evaluation system focusing on staffs, departments, and school performance. Medical faculty members are being asked to assume new academic duties for which they have received neither formal nor enough training, facing conflict and inefficiency. Faculty members do not believe that the evaluation processes fairly reward all of their efforts. However, the individuals are ultimately responsible for their own development. But academic leadership needs to have a clarified program for faculty and staff promotion and development, and it should be placed on a priority list and agenda. Positivism and encouragement, with faculty members as elites, are useful when considering Iranian's cultural and religious attributes.

### Fair and efficient management

The participants emphasized fairness, transparency, and documentation as an effective academic leadership principle. Department, school, and medical university leaders need to decide freely about their input, processes, and output territories. These domains of authorities constitute student acceptance, staff recruitment, budget expenditure and interpretation of centralized rules. Participants contended that academic leadership on the one hand, needs increased autonomy, authority, and emphasis on law-oriented management in order to convince management to be more effective and to be willing to take risk. They also need to act with autonomy about their processes and output with flexible and facilitated rules. On the other hand, academic leadership needs to have the capacities to use power resources such as legitimate, referent, reward, coercive, and expert power. However, academic leaders in Iran are most often challenged with imbalance between the breadth of their responsibilities and their authorities to manage. In addition, when considering their qualifications and selection criteria, academic leaders could only use their power of position (legitimate power), that is, the lowest level of leadership. There are many rigid and centralized rules and regulations at medical universities, schools, and departments that are not observed; both managers and staffs selectively use rules by lack of uncertainty avoidance (acceptance of assumed causes or explanations of a situation as fact to escape the discomfort associated with ambiguity or uncertainty), fair management, and rule of law.

### Climate of mutual trust and respect

The panelists shared the view that effective academic leadership needs to utilize communication skills, organizational culture, and shared values in order to fulfill mutual trust. Consequently, the interests of faculties, staffs, and leaders converge toward common organizational aims. Leaders should not only direct reciprocal communication, but also provide an effective communication network inside and outside of medical universities. Mutual trust and respect provide an appropriate context and move the organization toward individual and collective goal attainments. Organizational culture in Iranian medical universities does not show this mutual trust and respect. Usually, managers show little trust in academic staff and the reciprocal case is true as well. Because of the broadening of medical university's tasks in Iran, academic leadership has not taken the time and effort to build trust, integrity and the best interests of faculty members, staffs and stakeholders as well.

### Barriers and suggestions to effective academic leadership

**Figure 1:**
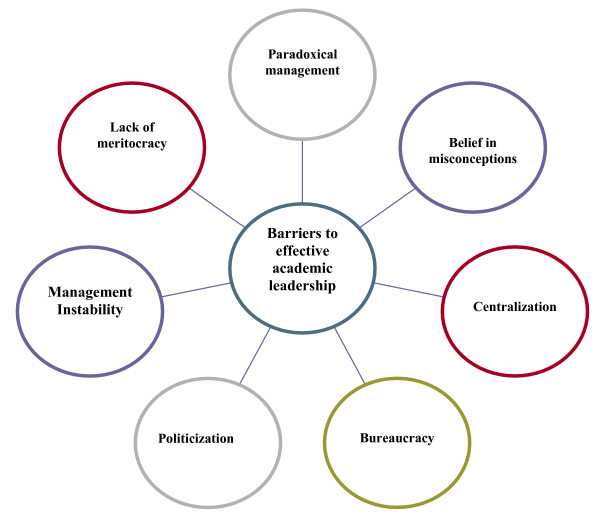
Barriers to effective academic leadership in Iran medical universities.

The views of the expert panel members about barriers for effective academic leadership were condensed into seven sub headings (See Figure 1.).

### Politicization, instability, and paradoxical management

The experts argued that politicization, instability and paradoxical management offer barriers to successful academic leadership at medical universities in Iran. Budgets, rules, structures, and even the recruitment of students are determined exclusively by the Ministry of Health. The university chancellors normally are determined by the Ministry. Others can also be influential like strong effective lobbies of some representatives of parliament, or influential parties. Instability of management is routine and occurs almost at all levels of academic leadership with recurrent cabinet and president changes. The effect of government and politicization of management in medical universities detract productivity and the quality of medical education at its foundation. Consequently, academic leadership usually experiences unstable management periods (1–4 years and rarely from some months to maximum 7 years). Paradoxical leadership is common because of lack of shared visions, goals, and strategies. Managers, who are elected by faculty members, are weakly accepted by chancellors and deans.

### Lack of meritocracy

The selection process of chancellor is confronted with too many factors. These factors could be mentioned as religious commitment and specific qualifications, as legal criteria and political considerations, as well as informal criteria. The most important participants in the selection process are the minister of Health and Medical Education, formal groups, Supreme Council of the Cultural Revolution, some members of parliament with diverse political tendencies, influential representatives at the provinces, some members of Board of Trustees, and some informal influential groups positioned inside and outside the medical university. In general, potential and qualified candidates to such high-level leadership do not easily accept this difficult responsibility because of potential power structures. Participants insisted on the establishment of a management database for medical universities in order to facilitate the selection process. For this purpose, a system for appointing medical managers should be employed, which supports training skills and assessment of aptitudes and interests.

### Centralization and bureaucracy

The expert panel was of the opinion that centralization, bureaucracy, and sever dependency to government with a strong hierarchical structure are important barriers to effective academic leadership. The hierarchical structure in medical universities spans from the top, The Ministry of Health and Medical education, to the heads of departments and faculty. An additional role legally accorded to the Minister of Health and Medical education includes acting as the chairman of the Board of Trustees of state medical universities. At medical universities, the Board of Trustees is the authority, seconded by the chancellor. This body has the highest power that directly influences the selection of other leadership positions, including heads of departments in some instances. Effective academic leadership needs to restructure itself toward a department-based system that decreases the multiple levels of decision-making, while increasing the department's autonomy in support of participatory decision-making. Arguably, the focus of the expert participants was on the levels of university and department, although, they sometimes complained about conflicts among schools and universities.

### Belief in misconceptions

Participant experts argued that belief in misconceptions have always been complicated to deal with in Iranian academic leadership. Belief in misconceptions such as "boss-oriented" mind, governmental ownership, fear from acceptance of responsibility, trouble of manipulation, could potentially exacerbate circumstances such as a severe willingness to uphold top-down decision-making, recognizing responsibility of reforms only upon government, necessity for power distance and hierarchy. There is a "voice" that always says: "why do I interfere with?" Highly experienced and qualified people usually have no motivation to accept managerial positions. The key to approaching restructuring is in managing as much as possible the efforts for internal changes, rather than being controlled entirely by external forces.

### Suggestion to effective academic leadership in Iran and developing countries

The expert panel concluded that performance-based management system followed by a payment-based system or financial allocation based on performance could be an appropriate strategy to motivate faculty members and academic leaders. This has been pilot tested at some Iranian medical universities, and early results are under assessment. But a willingness to support this kind of management is increasing. Iranian educational development centers (EDCs) are efficient in regard to staff, faculty, and academic leadership development. However, the medical academic leadership has not been making use of these centers or their capabilities at creating vision and strategic decision-making. Yet, their crucial roles on recent medical university reforms could not be ignored. Future reinforcement of EDCs could undoubtedly have an important role in teaching and research leadership. Iranian universities should be "free minded" and be able to decide upon their own academic and scientific governance. The medical university should be able to manage itself according to its specific visions, goals, and strategies. It should be able to choose students at post graduate course, with governmental, scientific, and logic stewardship and having strategic goals and visions with religious and cultural consideration. The movement toward mass education is essential with requirements of decreasing bureaucracy, volume of discipline subjects, study periods, lesson plans and educational programs. Policy makers and experts should consider crucial roles of religious and cultural issues to broaden higher education into society. In contrast they should consider also that cultural and faith issues should not negate scientific management and leadership in medical university governance.

## Discussion

The themes emerged from our study and deemed important for successful academic leadership are also representative of academic leadership in higher education in UK and Australia [13]. The most important issue discussed in detail was integrated academic leadership as a whole, as also proposed by the western world research. [13-24]. It is important for effective academic leadership to engage in sharing visions, goals, and strategies; to establish collaborative and transformational leadership and include full participation by delegating authority, and sharing responsibility among faculty; to help their development with a fair and effective reward system according to time and appropriate feedbacks; to increase meritocracy, which lack of has caused leadership to be based exclusively on leaders' authority or position (legitimate power); to gain sufficient autonomy and authority to direct resources toward achieving the overall objectives of medical education; and to have document-based strategies and plans.

To make academic leadership more effective, individual and organizational interests need to converge. This could be achieved through good communication and teamwork skills. Reciprocal communication and trust are important requirements and should be supported by faculty members and leaders. Academic leaders should induce trust and help faculty members to move towards individual and collective goal attainment. While many Iranian academic leaders presently have little trust in the academic staff and vice versa. Leaders have not taken the time and effort to build trust and integrity and think managing people is enough, and the literature shows that Iran is not an isolated case [16,20,25-27].

### Limitations

This study has some limitations that are worth mentioning. First, the participants do not cover the complete spectrum of medical education and management population. Second, NGT has a weakness that does not necessarily provide as valid information about attitudes and preferences as focus groups or individual interviews. We contend that an effort for proper understanding was made during the collection of our data.

### Directions for future research

This study is the first to analyze requirements for academic leadership in medical universities in Iran. This study can be replicated to include faculty members, students, and academic managers to enable comparisons to be drawn. Future research could also include the effect of organizational culture, values, and routines on academic leadership. This might shed a light on factors that cause academic culture and values to move in the direction they do following merger and to extend understanding of leadership requirements for effective academic leadership of mergers.

## Conclusion

The higher education management system in Iran has changed during recent years with the initiation of medical education reforms, including transitioning from a centralized to a decentralized university-based system. Because the medical education management system is only partially under the control of medical faculties, challenges to reform are in part the result of being confronted by the reality that capacity to change is only partially within the power of the institution itself. Indeed, medical universities may have not enough statutory powers to prove the needs to managerial system reform. Contrary to such selection measures, the complexity of academic leadership does not enhance its stature in Iran due to the merging of medical education and health services, where great responsibilities exists. Academic leadership is further exacerbated by problems such as lack of appropriate budget, supervision, and expansion of health issues. Arguably, selected academic leaders sometimes lack the required merits or appropriate qualifications, so they tend to be conservative. This is astounding, given that the position may often carry direct responsibilities for many hundreds of people, on a large budget, and the clinical quality. Perhaps these factors pose limitations among academic leaders and create unpleasant experiences about utilizing their authorities. Criteria supporting academic leadership have been delegated to politicization, informal groups, and external forces. In order to give medical leadership proper recognition and to encourage sensible career development, it is contended that a transparent and consistent approach must be adopted. Departments are the most important units supporting medical universities, holding the highest potential for transformation. Iranian academic leadership needs to restructure itself toward a department-based system that decreases the multiple levels of decision-making, while increasing the department's autonomy in support of participatory decision-making. The current school-based system has gained sympathy for its ability to reinforce individualism and boundaries between departments and university. Moreover, the department-based structure decreases the influence of a managerial layer and facilitates inter-departmental communications and networking. Such a structure could also decrease the phenomenon of power distance within the Iranian medical universities. Academic leaders should ponder the impact of a decision trying to enhance to trust and respect with all faculty members. If these issues are raised with every important decision, then the department, school, and even an entire university, should achieve the desired atmosphere of high levels of mutual trust and respect and increased academic productivity even in Iran.

## Competing interests

The author(s) declares that they have no competing interests.

## Authors' contributions

AB designed study, abstracted data, performed content analysis, interpreted the results, and drafted the manuscript. AS participated in initial study design, participated in study implementation, participated in content analysis and revised drafts. ZS participated in study implementation, participated in content analysis, and revised drafts. MB supervised the whole project, participated in initial study design, and revised the manuscript critically. IM interpreted reviewed the results, edited, and revised the manuscript critically.

## Pre-publication history

The pre-publication history for this paper can be accessed here:


